# Social–emotional architecture of adolescent success: a mixed-methods investigation of social–emotional learning pathways in Chinese secondary education

**DOI:** 10.3389/fpsyg.2025.1739807

**Published:** 2026-01-06

**Authors:** Lihua Qi, Wilson Cheong Hin Hong, Lifang Xue, Xiaoshu Xu, Wei Wei

**Affiliations:** 1 Faculty of Applied Sciences, Macao Polytechnic University, Macau, Macao SAR, China; 2 Faculty of Innovative Hospitality Management, Macao University of Tourism, Macau, Macao SAR, China; 3 Faculty of Creative Tourism and Intelligent Technologies, Macao University of Tourism, Macau, Macao SAR, China; 4 School of Foreign Studies, Wenzhou University, Wenzhou, China

**Keywords:** adolescents, China, collaborative for academic, digital resilience, online addictive behavior, social–emotional learning

## Abstract

**Introduction:**

This study investigates the relationship between social–emotional learning (SEL) competencies and online addictive behaviors among Chinese adolescents, using the Collaborative for Academic, Social, and Emotional Learning (CASEL) framework as a conceptual foundation.

**Methods:**

Drawing on a mixed-methods design, quantitative data were collected from 664 secondary students across 12 cities with a 63- item SEL questionnaire, and qualitative insights were obtained through semistructured interviews with 20 students.

**Results:**

Exploratory factor analysis identified a robust 12-factor structure that aligned with key SEL constructs; thematic analysis highlighted the interconnected nature of emotional regulation, relationship quality, and behavioral control in adolescents’ daily lives, while structural equation modeling confirmed significant pathways linking self-awareness, self-management, and social awareness to reduced online addictive behaviors, mediated by academic stress, interpersonal functioning, and mental health.

**Discussion:**

Findings underscore the protective role of SEL competencies in fostering digital resilience, academic engagement and psychosocial well-being, suggesting social and mental factors are greater determinants of problematic behavior than academic stress. This study provides one of the first empirical models linking SEL and digital behavior in a non-Western context, offering theoretical extensions to the CASEL model and practical guidance for school-based interventions. Implications for curriculum development and policy emphasize the urgent need to integrate SEL into national education strategies to support adolescent development in the digital era.

## 1 Introduction

Social and Emotional Learning (SEL) refers to the process through which individuals acquire and apply the knowledge, attitudes, and skills necessary to understand and manage emotions, establish and maintain positive relationships, make responsible decisions, and achieve personal and academic goals (Collaborative for Academic, Social, and Emotional Learning, 2020). As education systems around the world shift toward holistic models of development, SEL has been increasingly recognized as a critical component of student success (Jiang et al., 2024). It is not merely a supplementary set of interpersonal skills but a foundational element in fostering adaptive functioning in academic and social contexts (Belay and Dejene, 2024; Zins and Elias, 2007).

Globally, SEL has attracted substantial scholarly and policy attention. International research over the past two decades has shown that integrating SEL into school curricula can yield significant improvements in both academic performance and long-term mental well-being (Durlak et al., 2011; Melnick et al., 2017). Countries in North America, Europe, and parts of Asia have developed comprehensive intervention programs and assessment systems rooted in SEL principles, reflecting a global consensus on its transformative potential for educational reform (Eklund et al., 2018; Elliott et al., 2021).

Among the various theoretical models of SEL, the framework developed by the Collaborative for Academic, Social, and Emotional Learning (CASEL) is the most widely adopted. It organizes SEL into five interrelated competencies: self-awareness, self-management, social awareness, relationship skills, and responsible decision-making. The CASEL framework has served as the foundation for numerous evidence-based interventions and policy initiatives, particularly in Western contexts, where studies have consistently shown its effectiveness in enhancing students’ academic motivation, psychological resilience, and social functioning (Ross and Tolan, 2018; Vestad and Tharaldsen, 2022).

In the era of artificial intelligence, the significance of social and emotional learning (SEL) to the all-round development of students is becoming increasingly prominent, especially in China, where the intensity of education is extremely high. For a long time, Zhongkao and Gaokao have served as pivotal assessments determining students’ educational trajectories, significantly reinforcing a score-oriented approach to education and placing tremendous academic pressure on students. Chinese adolescents face a distinct set of challenges, including high academic stress, intense social competition, and an educational culture traditionally focused on cognitive achievement (Chen and Yu, 2022; Mou et al., 2022). According to a survey by Wang et al. (2024), the depression detection rate of fourth-grade students in China has reached 15.23%, among which the depression risk of the high academic pressure group is 1.78 times that of the low pressure group. In addition, more than 85% of adolescents do not meet the health standard of 60 min of physical activity per day, mainly due to heavy schoolwork (Yang et al., 2022). These stressors are linked to rising incidences of anxiety, relational conflict, and maladaptive behaviors such as Internet addiction (Chen et al., 2021; Kardefelt-Winther and UNICEF Office of Research - Innocenti, 2017; Yang et al., 2019). This reality shows that promoting SEL in an exam-oriented education environment is not only a supplement to ability development, but also a pressing need to enhance students’ psychological resilience and learning motivation.

Despite policy-level support for SEL in China’s national education agenda (Chen and Yu, 2022), empirical studies remain limited in scope and depth. Much of the existing research continues to rely on imported frameworks and instruments developed in Western contexts, where education landscapes are vastly different from that of China, let alone the absence of cultural considerations. Moreover, while some studies have examined the link between SEL and academic achievement, far less attention has been paid to its relationship with broader indicators such as mental well-being, social stress, and behavioral adjustment. Critically, there is a lack of systematic investigation into the factorial structure and cultural fit of the CASEL model within Chinese educational settings (Jiang et al., 2024; Ross and Tolan, 2018).

To address these gaps, this study aims to evaluate the applicability and structural validity of the CASEL five-competency framework among Chinese junior and senior high school students. Using a questionnaire survey and semi-structured interviews, the study assesses the internal structure of the CASEL model and examines how its five dimensions relate to students’ academic motivation, emotional state, peer-related stress, and patterns of Internet use. By developing a culturally responsive SEL assessment tool, the research seeks to promote more targeted interventions and policy recommendations that align with the realities of Chinese adolescents. The findings aim to inform the development of inclusive and supportive learning environments that nurture students’ comprehensive development and long-term well-being.

## 2 Literature review

### 2.1 Definition and importance of SEL in education

Social–emotional learning is defined as an integral part of education and human development, and refers to the process by which all youth and adults acquire and apply knowledge, skills, and attitudes to shape healthy identities, manage emotions, achieve personal and collective goals, feel and show empathy for others, build and maintain supportive relationships, and make responsible and caring decisions (Collaborative for Academic, Social, and Emotional Learning, 2021). SEL promotes educational equity and excellence through authentic school-family-community partnerships, learning environments and experiences characterized by trust and collaborative relationships, rigorous and meaningful curriculum and instruction, and ongoing assessment (Collaborative for Academic, Social, and Emotional Learning, 2021).

Extensive research has established that social and emotional functioning significantly contributes to students’ academic success, behavior regulation, and overall well-being (Ross and Tolan, 2018). SEL is particularly relevant in adolescence, where academic expectations often induce stress and undermine peer relationships and motivation (Bakken, 2018; Vestad and Tharaldsen, 2022).

Motivational beliefs have been identified as a key mediating factor in this relationship. Vestad and Tharaldsen (2022) found that promoting positive thinking, problem-solving skills, and a growth mindset through SEL interventions can enhance students’ ability to manage academic stress and support mental health. Similarly, Jiang et al. (2024) demonstrated that strengthening SEL competencies can indirectly boost academic achievement by reinforcing students’ motivational beliefs. Their findings further showed that students with higher SEL competency profiles exhibit more adaptive academic outcomes.

SEL has also shown heightened effectiveness among adolescents with behavioral challenges or low self-efficacy. Chen et al. (2021) confirmed that school-based SEL programs can significantly support psychosocial development and mitigate behavioral risks. At the policy level, organizations such as the OECD have advocated for SEL as a foundational skill set for future-ready learners, emphasizing emotion regulation and social interaction (Chen and Yu, 2022; Denham, 2006).

Recent advancements also suggest that technological enhancements can support SEL. Zong and Yang (2025) reported that AI-integrated SEL programs improved students’ emotional regulation, attention, and academic engagement. These findings collectively underscore the importance of integrating SEL into adolescent education, especially under conditions of academic stress and behavioral risk.

### 2.2 Multifaceted impacts of SEL on students’ development and adjustment

A growing body of research has demonstrated strong correlations between students’ social–emotional competence and their academic development. In China, studies have confirmed that SEL positively predicts academic performance, engagement, and peer relationships. For instance, Wang (2022) found that SEL competence remains relatively stable during early adolescence and contributes positively to academic outcomes. Similarly, Wang et al. (2019) highlighted SEL as a key factor influencing students’ learning participation and interpersonal skills. Jiang et al. (2024) further identified four distinct SEL profiles among Chinese adolescents and found that higher SEL levels were associated with stronger academic adaptability. Internationally, the Strengths and Difficulties Questionnaire (SDQ) has been employed to examine similar links, particularly in studies with smaller samples (Goodman, 1997).

The psychological benefits of SEL are also well-documented. Durlak et al. (2011) emphasized that enhancing emotional regulation and self-awareness through SEL helps reduce anxiety, depression, and behavioral issues. In Norway, Vestad and Tharaldsen (2022) demonstrated that SEL-based training improved students’ emotional resilience to academic stress. In China, Chen et al. (2021) found that CASEL-based programs can foster psychosocial development, particularly for students with low self-efficacy. Jiang et al. (2024) similarly reported that students with higher SEL competence showed significantly better mental health outcomes. Teacher support also plays a critical role; emotional support from educators has been found to enhance students’ emotional regulation and psychological well-being (Xie et al., 2022).

Interpersonal development is another essential outcome of SEL. Wang et al. (2019) emphasized its role in improving the quality of adolescent peer interactions. Xie et al. (2022) found that teachers’ emotional labor strategies—such as deep or surface acting—significantly influence students’ SEL development. Moreover, teacher support for students’ autonomy, competence, and relatedness has been shown to predict the growth of all five CASEL competencies (Wang et al., 2025). Tools such as the BERS-2 are commonly used to assess interpersonal skills and emotional expression from the perspectives of students, teachers, and parents (Buckley and Epstein, 2004; Epstein et al., 2004).

SEL has also been linked to the reduction of high-risk behaviors, including problematic Internet use (PIU) (Chen et al., 2024). Chen et al. (2021) found that while bullying correlates positively with PIU, higher SEL ability can serve as a protective factor. However, in certain cases, high SEL may also intensify the link between stress and maladaptive behaviors, indicating a more complex interaction between emotional regulation and external pressures. The Communities That Care (CTC) framework similarly underscores the importance of multi-level support in preventing adolescent risk behaviors (Achenbach and Rescorla, 2014; Haggerty et al., 2010).

### 2.3 Assessment tools of SEL

As SEL research has expanded, the development of valid and reliable assessment tools has become increasingly sophisticated. A variety of instruments have been employed internationally to capture students’ social–emotional competencies across diverse contexts. These include the Comprehensive School Climate Inventory (CSCI) (Walsh and Weiser, 2024) and the Developmental Asset Profile (DAP) (Zaker et al., 2024), which assess school climate and developmental supports.

Strengths-based tools such as the Devereux Student Strengths Assessment (DESSA) (LeBuffe et al., 2018), the Behavioral and Emotional Rating Scale (BERS-2) (Buckley and Epstein, 2004), and the Social Skills Improvement System (SSIS) (Gresham and Elliott, 2008) are widely used in school-based interventions and longitudinal studies. The ASEBA system (Achenbach and Rescorla, 2014), widely adopted in clinical and educational psychology, offers multidimensional data that supports robust SEL evaluation.

Importantly, there is a compelling need for theory-driven assessment instruments to enhance the psychometric rigor and practical relevance of SEL research (Haggerty et al., 2010). As the field continues to evolve, culturally and developmentally responsive tools remain essential for capturing the complexity of SEL across diverse populations.

### 2.4 The CASEL competency framework

The CASEL five-core competency framework has become a foundational model in global Social and Emotional Learning (SEL) research. As a strengths-based framework, it has shaped the design of numerous interventions and informed both theoretical and empirical inquiries (Eklund et al., 2018; Elliott et al., 2021; Ross and Tolan, 2018). The framework consists of five interrelated competencies: self-awareness, self-management, social awareness, relationship skills, and responsible decision-making—each targeting a critical dimension of emotional regulation, interpersonal functioning, and ethical reasoning (Elliott et al., 2021; Ross and Tolan, 2018).

Beyond its conceptual clarity, the CASEL model also aims to promote educational equity by embedding SEL across age groups, disciplines, and sociocultural settings (Collaborative for Academic, Social, and Emotional Learning, 2020). It has been extensively applied in Western educational systems, contributing to a more systematic understanding and measurement of SEL (Ross and Tolan, 2018; Thomas et al., 2022). However, empirical validation of its multidimensional structure remains limited in non-Western contexts, particularly in adolescence-focused research. Prior studies have predominantly concentrated on younger learners and often overlooked broader adolescent outcomes such as academic motivation, mental health, behavioral adjustment, and peer-related stress (Belay and Dejene, 2024; Ross and Tolan, 2018).

Preliminary efforts have been made to test the model’s cross-cultural applicability. Jiang et al. (2024), for instance, found that the five competencies demonstrated meaningful structural validity in a sample of Chinese secondary school students, offering initial support for its relevance in Chinese contexts. Yet, challenges remain. Compared to the well-established international body of SEL literature, Chinese SEL research is still in its formative stages. It is characterized by limited theoretical localization, over-reliance on imported models and tools, a lack of intervention-based studies, and narrow research perspectives (Jiang et al., 2024). These gaps underscore the pressing need for culturally responsive assessment tools and intervention frameworks adapted to Chinese adolescents’ social, academic, and emotional realities.

The current study aims to validate the CASEL framework in a Chinese adolescent sample and explore its associations with academic pressure, relational stress, mental well-being, academic performance, behavioral regulation, and digital escape behaviors. Specifically, this study addresses the following research questions:

(1) How well does the CASEL model fit the data collected from junior middle school students in China?
(2) What are the relationships among the five SEL competencies in this population?
(3) How do these competencies relate to students’ responses to academic and relational pressure, well-being, performance, behavioral adjustment, and problematic Internet use?

## 3 Methods

### 3.1 Research design

This study adopted a mixed-methods design to explore the complex relationships between adolescents’ social–emotional competencies and their academic, psychological, and behavioral outcomes. By integrating quantitative and qualitative approaches, the design allowed for both generalizable patterns and in-depth contextual understanding (Creswell and Creswell, 2017).

The quantitative strand utilized questionnaires to assess key SEL domains, including mental health, academic strategies, interpersonal functioning, and risk behaviors. While, the qualitative strand employed semi-structured interviews to gain nuanced insights into students’ subjective experiences with emotional regulation, academic pressure, and social relationships. The combination of these methods enabled a comprehensive examination of both measurable outcomes and the underlying mechanisms shaping adolescent development.

### 3.2 Sample and sampling

Convenience sampling was applied in 20 secondary schools across 12 cities, ranging from metropolitan (Shanghai, Beijing, Macao), medium-sized (Wenzhou, Ningbo, Taizhou), to lesser-known cities (Huzhou, Jinhua, Lishi) in China. A total of 664 students from Grades 7 to 12 voluntarily participated with their parents’ consent, representing a diverse adolescent population. This sample size was determined sufficient for detecting medium effect sizes (*f*^2^ = 0.15) with 95% power per G*Power analysis. Data were inspected for excessively short or straight-lining responses and outliers, leaving a final 592 valid responses.

Participants ranged in age from 13 to 16 years (*M* = 14.2), with a slightly higher proportion of female students (51.5%). Grade distribution was as follows: Grade 8 (58.4%), Grade 7 (14.8%), Grade 11 (12.7%), Grade 10 (8.3%), Grade 12 (4.8%), and Grade 9 (1.1%). The lower participation in Grades 9 and 12 is likely due to academic demands associated with the Zhongkao and Gaokao entrance examinations.

### 3.3 Instruments

Two instruments were used in this study: a self-developed questionnaire and a semi-structured interview protocol, both aligned with the SEL framework proposed by Ross and Tolan (2018).

The questionnaire consisted of 60 items across 12 dimensions (Mental Health, Responsible Decision-Making, Academic Strategies, Relationship Quality, Online Addictive Behavior, Self-Awareness, Social Awareness, Academic Pressure, Self-Management, Creating Relationships, and Reliability), measuring constructs such as mental health, self-management, responsible decision-making, academic strategies, relationship quality, and online addictive behaviors. Items were rated on a 5-point Likert scale (1 = Strongly Disagree to 5 = Strongly Agree). The English version was translated and back-translated into Chinese to ensure conceptual equivalence. Reliability analysis showed acceptable to good internal consistency (Cronbach’s α = 0.53–0.86). Subsequent confirmatory factor analysis supported construct validity (χ^2^ = 23233.855, df = 1,770, CFI = 0.82, RMSEA = 0.064), to be elaborated later.

The semi-structured interview protocol, on the other hand, was developed based on the same SEL framework and informed by preliminary analysis of quantitative findings. The protocol contained 15 open-ended questions exploring four domains: emotional regulation, academic stress, peer relationships, and technology use. The prompting technique was used to avoid leading interviewees to provide certain responses. Example questions included: “Can you describe a recent situation where you had to manage strong emotions?” and “How do you balance academic demands with other aspects of your life?” The protocol was reviewed by three experts in adolescent development and piloted with four students to ensure clarity and developmental appropriateness. Interviews were conducted in Chinese by trained interviewers with backgrounds in educational psychology.

### 3.4 Data collection

Quantitative data were collected from 664 students between December 2024 and March 2025. Data collection was slow as the target participants were not adults, and extra time was taken to ensure research ethics (e.g., obtaining parental consent). Quota sampling was initially planned but was found to be challenging for this reason and across-city collections. Thus, convenient sampling was conducted. Volunteering students completed the questionnaire under teachers’ supervision.

The qualitative phase happened simultaneously, 20 participants (13 males, 7 females) were purposefully selected using maximum variation sampling across grade levels, gender, and questionnaire responses (Kvale, 2009). Theoretical saturation was safeguarded with 2 participants not offering new insights. Interviews conducted in private school rooms lasted 25–35 min each. Considering participants might not like to share certain information in front of their parents, interviews were conducted in the absence of parents, but parental consent included audio-recording, and transcribed verbatim with identifiers removed.

### 3.5 Data analysis

Quantitative data were analyzed using SPSS28. After data cleaning and assumption testing, descriptive statistics were calculated for all variables. Inferential analyses included correlation analysis, multiple regression, and structural equation modeling to examine relationships between SEL competencies and outcome variables.

Qualitative data were analyzed using thematic analysis following Braun and Clarke’s (2006) six-phase framework. A three-stage coding procedure was adopted. During open coding, recurring patterns were identified across participants’ narratives, including strategies for emotion recognition, emotional regulation, self-management, and interpersonal understanding. Example codes included nonverbal cue interpretation, physical activity for emotional control, time management behaviors, and perceived links between emotional states and academic performance. In the subsequent phases, themes were refined and organized through iterative comparison and discussion. Two researchers independently coded the data and resolved discrepancies through dialog, achieving an inter-coder reliability rate above 90%. This ensured analytical rigor and consistency. The qualitative findings complemented the quantitative results by providing nuanced insights into the ways social–emotional competencies influence students’ academic and social development.

## 4 Results

### 4.1 Exploratory factor analysis (QUAN)

An Exploratory Factor Analysis (EFA) was conducted to examine the underlying factor structure of the measurement instrument. Prior to analysis, the Kaiser–Meyer–Olkin (KMO) measure of sampling adequacy was calculated at 0.913, exceeding the recommended threshold of 0.80 and indicating excellent suitability of the data for factor analysis. Bartlett’s Test of Sphericity was significant (χ^2^ = 23233.855, df = 1,770, *p* < 0.001), confirming that the correlation matrix was appropriate for factor extraction.

The EFA was performed using Principal Component Analysis (PCA) with Varimax rotation. The analysis extracted 12 factors with eigenvalues greater than 1.0, accounting for 67.8% of the total variance. These factors corresponded closely to the theoretically defined constructs: Mental Health, Responsible Decision-Making, Academic Strategies, Relationship Quality, Online Addictive Behavior, Self-Awareness, Social Awareness, Academic Pressure, Self-Management, Creating Relationships, and Reliability.

Two items were excluded due to low factor loadings (<0.50)—one from the Self-Awareness subscale and one from the Interpersonal Pressure subscale. All remaining items loaded strongly on their intended factors, with loadings ranging from 0.53 to 0.86, indicating robust construct validity. The rotated component matrix revealed minimal cross-loadings, providing additional support for the discriminant validity of the factor structure (see Table 1).

**Table 1:** Factor loading matrix.

Factor	Loading
Self management	0.565–0.758
Self-awareness	0.743–0.808
Social awareness	0.663–0.798
Creating relationships	0.716–0.790
Relationship quality	0.777–0.842
Responsible decision making	0.661–0.855
Academic strategies	0.686–0.821
Reliability	0.692–0.721
Academic pressure	0.756–0.832
Mental health	0.657–0.808
Interpersonal pressure	0.533–0.761
Online addictive behavior	0.615–0.815

### 4.2 Thematic analysis (QUAL)

After confirming the validity and reliability of the constructs, thematic analysis was performed in an attempt to understand the underlying relationship among the constructs. The analysis revealed several interrelated dimensions of adolescents’ social–emotional experiences, illustrating how emotional competencies influence academic, behavioral, and interpersonal outcomes. Themes were developed inductively based on participant narratives and are supported by representative quotes (see Table 2).

**Table 2:** Coding analysis.

Theme	Description	Key codes	Examples
Emotional awareness and recognition	How individuals recognize and understand emotions in others	Observation of nonverbal cues, direct communication, active listening	“看别人的行为以及神态” (Observing others’ behaviors and expressions)
Emotional regulation strategies	Methods used to manage emotional states	Physical activities, entertainment/distraction, Social support	“踢足球和听音乐” (Playing soccer and listening to music)
Self-management abilities	Ability to regulate behavior and time	Time management, self-control, behavioral monitoring	“用计划表安排作业和休息” (Using schedules for homework and rest)
Emotion-academic performance connection	How emotions influence learning	Negative emotions decrease efficiency, positive emotions enhance learning	“情绪不好就学着很累” (Bad mood makes studying exhausting)
Social understanding and relationships	Understanding of relationship quality	Trust and respect, mutual support, communication	“信任对方能保守秘密” (Trust others to keep secrets)
Interconnections between life domains	How different life areas influence each other	Emotion-behavior-social-academic pathway	“社交影响情感, 情感影响学习” (Social affects emotions, emotions affect learning)
Internet usage patterns	Technology use habits	Digital resilience, learning use, time limitations	“每周离校时追更新的漫画” (Following manga/anime updates weekly)
Support systems	Resources that help students develop	Family support, peer monitoring, alternative activities	“父母, 朋友的监督与自我监督” (Supervision from parents, friends and self)

Emotional awareness emerged as a foundational competency shaping emotional responses and interpersonal behavior. Participant #2 described identifying others’ emotions by observing nonverbal cues, while Participant #17 elaborated that recognizing a peer’s distress prompted her to initiate a supportive response. This recognition-to-regulation progression was consistently reflected across accounts.

Self-management abilities were strongly linked to behavioral regulation and academic engagement. Participant #10 reported using detailed personal plans to manage study time and control technology use. Similarly, Participant #4 attributed effective behavioral regulation to strong self-management skills across both academic and social contexts.

The connection between emotion and academic performance was another recurring theme. Participant #5 stated that negative emotional states made studying feel exhausting and impaired academic performance, while Participant #10 suggested that, for some, negative emotions could occasionally increase focus and efficiency—highlighting individual variability in emotional responses.

Support systems were identified as key factors in promoting emotional regulation and behavioral control. Participant #9 emphasized the combined role of parental, peer, and self-monitoring in reducing problematic internet use. Interestingly, some students also viewed technology itself as a form of emotional support. For example, Participant #6 shared that following manga and anime updates helped him decompress after school, suggesting that when used appropriately, technology can serve as a coping mechanism.

Students also demonstrated a nuanced understanding of interconnections between life domains. Participant #8 explained how social interactions affect emotions, which in turn influence learning outcomes. Participant #19 described how academic setbacks, such as poor test performance, led to emotional distress that impacted other areas of life. These accounts underscore students’ awareness of the dynamic and reciprocal nature of emotional, academic, and social experiences.

#### 4.2.1 Key relationships among themes

Important relationships among the themes were identified. Emotional recognition directly facilitated supportive interactions, with participants identifying others’ emotional states through nonverbal cues as a pathway to meaningful connections. Peer relationships served dual roles as emotional resources during academic challenges and sources of social complexity. These relationships constituted significant elements in students’ emotional support systems.

Technology functioned as both connector and barrier in relationships. Students valued digital platforms for maintaining friendships while acknowledging how excessive use undermined face-to-face interactions. Family relationships provided essential emotional scaffolding. Participants frequently cited parental guidance for managing emotions and social challenges, demonstrating continued family influence alongside growing peer importance.

Relationship quality showed clear bidirectional connections with academic performance and emotional wellbeing. Overall, students articulated how relationship difficulties affected academic engagement and emotional states, highlighting healthy relationships’ centrality to adolescent development, more so than factors directly related to studies (e.g., workload).

### 4.3 Structural equation modeling (QUAN)

Based on the interview results and an established framework (Ross and Tolan, 2018), we attempted to verify the relationship of the quantitative SEL constructs using Structural Equation Modeling (SEM). Prior to model estimation, multicollinearity diagnostics were conducted. Tolerance values ranged from 0.62 to 0.78, and Variance Inflation Factor (VIF) values from 1.17 to 1.87, all falling within acceptable limits (Tolerance > 0.10; VIF < 5), indicating no significant multicollinearity issues.

#### 4.3.1 Model development

This study employed a full-information structural equation modeling (SEM) framework that simultaneously estimated both measurement and structural components. Latent constructs were defined using observed indicators identified through the preceding exploratory factor analysis (EFA), and the predefined factor structure was validated with confirmatory factor analysis (CFA). The model was integrated into the overall SEM, and all factor loadings, as well as structural paths, were estimated concurrently. This approach allowed for a comprehensive assessment of construct validity and the directional relationships among variables.

The model was refined through an iterative, theory-driven process. Initial specifications (5 SEL constructs) was based on Ross and Tolan (2018), additional constructs were step-wisely incorporated to capture the multidimensional nature of adolescent development. Pathways were added or modified according to previous findings and empirical contribution to model fit. This approach ensured that the final model remained both conceptually grounded and statistically robust.

#### 4.3.2 Model fit and interpretation

The final five-level structural model demonstrated acceptable fit. Key indices included: Incremental Fit Index (IFI = 0.83), Tucker-Lewis Index (TLI = 0.81), Comparative Fit Index (CFI = 0.82), and Root Mean Square Error of Approximation (RMSEA = 0.064), all within or above conventional thresholds for model adequacy (Hu and Bentler, 1999; MacCallum et al., 1996).

**Figure 1:**
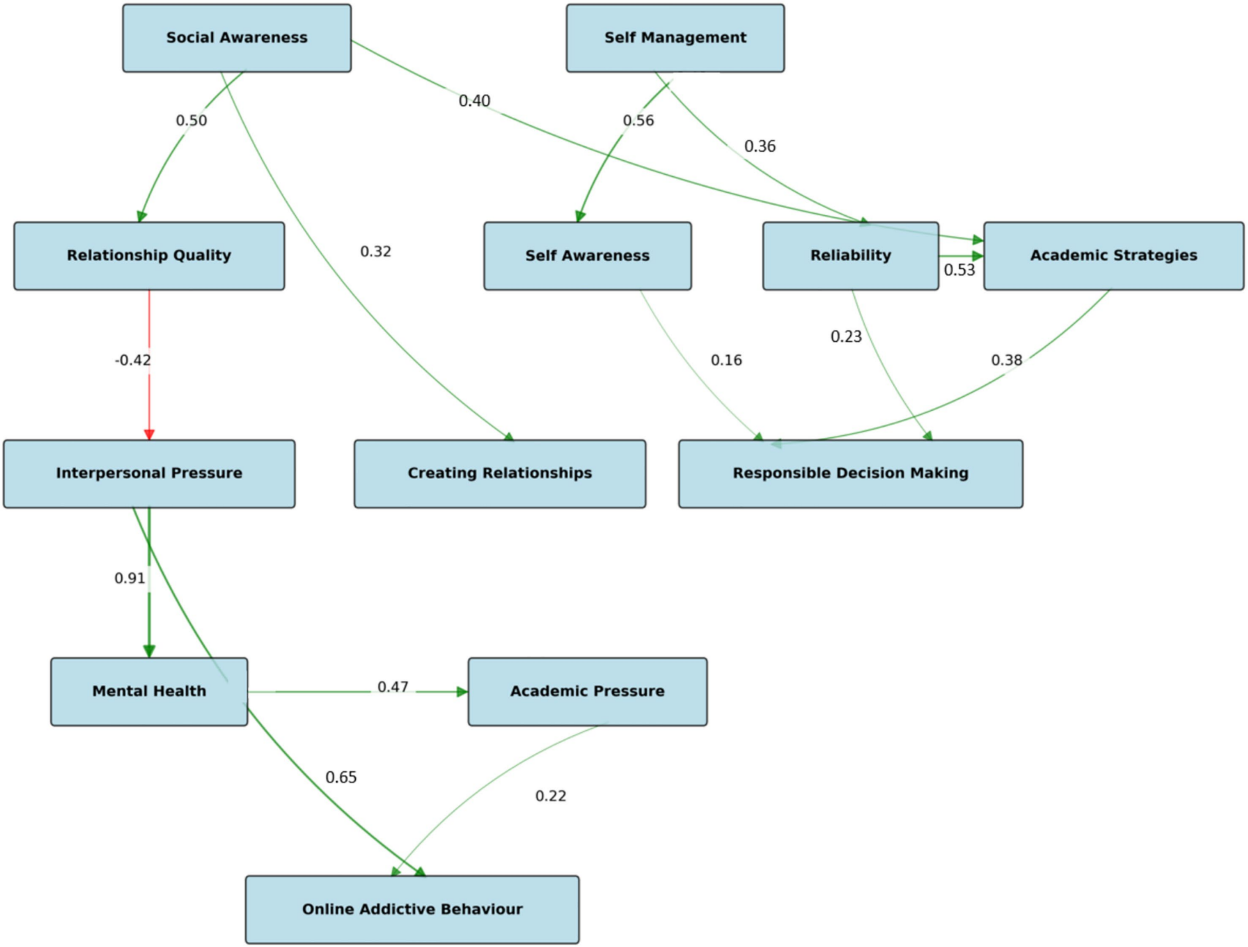
Model structure. *Note*: *p* < 0.001; Green arrow pointing to the right on a white background = positive relationship; Red arrow pointing to the right on a white background = negative relationship.

The final model showed a five-level structure (Figure 1). Given the model’s complexity—encompassing 12 constructs and multiple direct and indirect pathways—these indices reflect a satisfactory balance between theoretical richness and statistical performance (Marsh et al., 2004). The model effectively captured the interconnections among SEL competencies, academic strategies, interpersonal relationships, and behavioral outcomes, providing a comprehensive framework for understanding the structural dynamics underlying adolescent development.

See Table 3 for detailed SEM results and Figure 1 for a visual representation of the final model.

**Table 3:** Path relationship table of structural equation model.

Pathway	Estimate	S. E.	C. R.	*P*
Academic pressure → online addictive behavior	0.22	0.056	3.927	***
Academic strategies → responsible decision making	0.377	0.052	7.28	***
Interpersonal pressure → mental health	0.907	0.058	15.629	***
Interpersonal pressure → online addictive behavior	0.648	0.061	10.676	***
Mental health → academic pressure	0.472	0.044	10.715	***
Relationship quality → interpersonal pressure	−0.422	0.053	−7.987	***
Reliability → academic strategies	0.531	0.058	9.086	***
Reliability → responsible decision making	0.233	0.056	4.154	***
Self awareness → responsible decision making	0.163	0.033	4.92	***
Self management → self awareness	0.564	0.065	8.661	***
Self management → reliability	0.363	0.054	6.758	***
Social awareness → academic strategies	0.402	0.046	8.761	***
Social awareness → creating relationships	0.322	0.059	5.462	***
Social awareness → relationship quality	0.498	0.055	9.105	***

##### 4.3.2.1 Level 1: predictor variables

At the top level, Social Awareness and Self Management functioned as exogenous variables, serving as primary predictors in the model. These variables were not predicted by other variables within the model but exerted significant influence on downstream constructs.

##### 4.3.2.2 Level 2: primary mediators

The second level consisted of Relationship Quality (β = 0.50, *p* < 0.001), Self Awareness (β = 0.56, *p* < 0.001), Reliability (β = 0.36, *p* < 0.001), and Academic Strategies (β = 0.40, *p* < 0.001 from Social Awareness; β = 0.53, *p* < 0.001 from Reliability). These variables served as primary mediators, receiving direct effects from the exogenous variables and transmitting these effects to lower-level constructs.

##### 4.3.2.3 Level 3: secondary mediators

The third level included Interpersonal Pressure (β = −0.42, *p* < 0.001), Creating Relationships (β = 0.32, *p* < 0.001), and Responsible Decision Making (β = 0.23, *p* < 0.001 from Reliability; β = 0.38, *p* < 0.001 from Academic Strategies; β = 0.16, *p* < 0.001 from Self Awareness). These variables functioned as secondary mediators, receiving effects from the primary mediators and transmitting them to subsequent levels.

##### 4.3.2.4 Level 4: tertiary mediators

The fourth level consisted of Mental Health (β = 0.91, *p* < 0.001) and Academic Pressure (β = 0.47, *p* < 0.001), which served as tertiary mediators in the model. These variables were influenced by the secondary mediators and, in turn, affected the outcome variable.

##### 4.3.2.5 Level 5: outcome variable

At the bottom level, Online Addictive Behavior served as the primary outcome variable, receiving effects from both Interpersonal Pressure (β = 0.65, *p* < 0.001) and Academic Pressure (β = 0.22, *p* < 0.001).

#### 4.3.3 Notable pathways

The model revealed several important pathways. The strongest direct effect was observed between Interpersonal Pressure and Mental Health (β = 0.91, *p* < 0.001), suggesting that pressure in interpersonal relationships strongly impacts mental health outcomes. Additionally, Interpersonal Pressure demonstrated a substantial direct effect on Online Addictive Behavior (β = 0.65, *p* < 0.001), indicating that relationship pressures may contribute significantly to problematic online behaviors.

The model also highlighted the protective role of Social Awareness, which positively influenced Relationship Quality (β = 0.50, *p* < 0.001), Academic Strategies (β = 0.40, *p* < 0.001), and Creating Relationships (β = 0.32, *p* < 0.001). Similarly, Self Management showed positive effects on Self Awareness (β = 0.56, *p* < 0.001) and Reliability (β = 0.36, *p* < 0.001).

Notably, Relationship Quality had a negative effect on Interpersonal Pressure (β = −0.42, *p* < 0.001), suggesting that higher relationship quality may serve as a buffer against perceived interpersonal pressures.

This five-level structural model provides a comprehensive framework for understanding the complex interrelationships among social–emotional competencies, academic factors, and online behavioral outcomes.

## 5 Discussion and conclusions

### 5.1 General discussion

This study extends the conceptual reach of the CASEL social–emotional learning (SEL) framework by examining its applicability to Chinese adolescents and exploring how SEL competencies influence academic performance, mental health, interpersonal stress, behavioral regulation, and online addictive behaviors. Grounded in our interviews and SEM model, the results confirm the multidimensional and interconnected nature of these constructs and offer new insights into the mechanisms through which SEL competencies shape developmental outcomes.

The model demonstrated strong psychometric properties and theoretical coherence. Most pathways among SEL dimensions, academic and behavioral outcomes, and online behaviors were statistically significant and directionally consistent with the literature. One exception was the unexpected negative relationship between relationship quality and interpersonal stress, which may reflect unique cultural dynamics within Chinese peer relationships, such as the pressure to maintain harmony or the fear of “losing face,” which is closely tied to young people’s strong self-esteem and desire to belong to social groups (Yen et al., 2024). Nonetheless, the overall model supports the cross-cultural applicability of the CASEL framework and reinforces its value in non-Western educational contexts.

Notably, the construct of relationship skills emerged as multidimensional rather than singular (Ross and Tolan, 2018). Instead of loading onto a single latent factor, items clustered around two subdimensions—“creating relationships” and “relationship quality”—highlighting an important developmental distinction. While the former involves initiating connections, the latter reflects the depth and maintenance of those relationships. This differentiation aligns with developmental research suggesting that adolescents’ early life experiences and evolving social environments influence not only their ability to form relationships but also the quality of those relationships over time (Berry and O’Connor, 2010; Ross and Tolan, 2018). The split may also reflect the relatively intricate relationships in the Chinese culture (Yen et al., 2024).

Our findings reveal two distinct pathways through which SEL competencies influence adolescent outcomes. The first pathway, grounded in Social Cognitive Theory (Bandura, 2001), demonstrates how social awareness functions as a significant predictor of academic strategy use, mediated by relationship quality and interpersonal stress. The corroborated findings explain how individuals’ ability to interpret social cues informs their behavioral regulation and stress responses. High levels of social awareness buffer academic pressure by promoting supportive relationships and reducing relational conflict. This social pathway aligns with recent empirical findings highlighting the cascading effects of social stress on academic behavior (Mou et al., 2022) and supports the emphasis on SEL’s role in improving the quality of adolescent peer interactions (Wang et al., 2019).

The second pathway, aligned with the Goal-Directed Behavior Model (Locke and Latham, 2006), shows how intrapersonal competencies—particularly self-awareness and self-management—foster effective academic strategies and responsible decision-making. As adolescents gain clearer perceptions of their capabilities through self-management routines, they adopt more systematic academic strategies, improving performance outcomes (Guo et al., 2016; Zimmerman, 2000). SEL interventions promoting positive thinking and problem-solving skills enhance students’ ability to manage academic stress (Vestad and Tharaldsen, 2022). These support earlier findings that SEL competencies reinforce motivational beliefs that support academic achievement (Jiang et al., 2024).

Notably, while academic stress can directly cause online addictive behavior, supporting existing findings (Jasper et al., 2023; Mun and Lee, 2024; Yang et al., 2023), we further strengthen earlier suggestions that much more important determinants of online addiction were relationship issues and mental states of the student (Mun and Lee, 2024; Yang et al., 2023). High-quality interpersonal relationships emerged as a protective factor moderating the risk of internet addiction. In other words, the same amount of academic pressure for students with positive mental states and healthy relationships and those in poorer mental states and relationships could be vastly different. Hence, as Durlak et al. (2011) suggested, enhancing students’ emotional regulation and social skills is vital in reducing behavioral issues. Interestingly, though, our interview findings indicated that adolescents may resort to digital platforms to regulate stress and fulfill unmet emotional needs. While purposeful and moderate use of digital tools can serve as a constructive outlet to support emotional well-being (Wu et al., 2016), if excessive digital use is left unmonitored, they may evolve into habitual avoidance responses (Marciano et al., 2022).

In sum, this study advances our understanding of SEL in the context of Chinese adolescent development; it comprehensively describes two pathways—social and intrapersonal—through which SEL influences academic performance, mental health, and online behavior. The findings underscore the importance of a holistic approach to adolescent education that integrates SEL competencies into curriculum design, teacher training, and educational policy, especially as academic pressure continues to intensify and society becomes increasingly complex.

### 5.2 Theoretical contributions

This study makes several contributions to SEL theory. First, by modeling the indirect pathways through which SEL competencies influence adolescent outcomes, we extend beyond the direct-effects approach that has dominated previous research (e.g., Durlak et al., 2011). Our findings reveal a more complex, mediated relationship between SEL competencies and developmental outcomes, highlighting the intervening roles of mental health, self-perception, and interpersonal stress. This nuanced understanding enriches the explanatory depth of the SEL framework and provides a more comprehensive theoretical model for future investigations.

Second, our identification of the social and intrapersonal paths suggest connections between social skills through the lens of Social Cognitive Theory (Bandura, 2001) and self-management via the Goal-Directed Behavior Model (Locke and Latham, 2006) in achieving academic outcomes or alternative behaviors. Further, our finding that relationship skills comprise two distinct dimensions—creating relationships and maintaining relationship quality—refines the conceptualization of this core SEL competency. This multidimensionality aligns with developmental research suggesting that relationship formation and maintenance involve different psychological processes (Ross and Tolan, 2018), thereby enhancing the theoretical precision of the CASEL framework, and informing distinct mechanisms that influence adolescent development.

### 5.3 Practical implications

Our findings offer several actionable implications for educational practice and policy. First, schools should embed SEL competencies systematically across curricula rather than treating them as isolated programs. Integrating positive thinking, problem-solving skills, and growth mindset into academic instruction can enhance students’ ability to manage stress while supporting achievement (Vestad and Tharaldsen, 2022; Cavioni et al., 2024). This is especially true in learning-intensive secondary school environments in China. Interestingly, results also indicate that academic stress itself is not a major determinant of problematic behaviors, raising questions about the necessity of low-stress “happy schooling” (Salmon, 2016), a typical Western educational philosophy. What is more important is that teachers should incorporate emotional regulation, self-awareness, and interpersonal skills into daily lessons, with particular attention to students showing signs of emotional distress or academic disengagement. Crucially, while many existing programs focus primarily on intrapersonal skills like self-management, our findings highlight the equal importance of social awareness and relationship quality. Comprehensive SEL initiatives should therefore balance individual competency development with strategies for fostering positive peer relationships and supportive classroom environments (Wang et al., 2019).

Then, schools should develop integrated approaches to digital literacy and SEL. Our findings regarding the relationship between emotional regulation and online behavior suggest that teaching responsible technology use should extend beyond technical skills to include emotional awareness and healthy coping strategies. This integrated approach can help students leverage digital tools constructively while minimizing risks of problematic use, supporting previous recommendations (Wu et al., 2016). As society—online and offline—becomes increasingly complex, there is a need to expand SEL programs through comprehensive school-family-community support systems to enhance the effectiveness of SEL initiatives (Thomas et al., 2022).

Certainly, appropriate measures can only be taken with assessments that capture both academic and social–emotional developmental aspects, with considerations of cross-cultural applicability. By monitoring SEL competencies alongside traditional academic metrics, schools can identify students needing additional support and evaluate the need/effectiveness of interventions (Jiang et al., 2024).

### 5.4 Limitations and future research

Despite this study’s contributions, limitations exist. First, its cross-sectional design limits the ability to capture developmental changes in SEL competencies and their long-term impact on adolescent behavior; future longitudinal research is needed to address this gap. Second, the reliance on self-reported data introduces potential biases, including social desirability effects and misinterpretation of abstract terms and Likert scale gradations. Incorporating multi-informant assessments (e.g., from teachers or parents) and using more nuanced response formats could improve measurement accuracy and validity (Elliott et al., 2021). Third, addictive Internet behavior is used as a proxy for problematic behavior in this study, but we acknowledge Internet addiction cannot be generalized to other forms of problematic behavior (e.g., self-harming). Such, nevertheless, is a highly sensitive topic that cannot be supported by our methods. Future studies can use non-self-reporting means to gage sensitive problematic behaviors, such as applying psychological experiments like the Implicit Association Test (Karpinski and Hilton, 2001).

In future research, the integration of intelligent tutoring systems (ITS) with emotion recognition and regulation capabilities offers a promising avenue for enhancing the real-time assessment of learners’ affective states. By leveraging sentiment analysis within ITS environments, researchers can investigate how emotional dynamics interact with the development of SEL competencies and academic engagement (Ben Ammar et al., 2010). This line of inquiry not only improves the precision of SEL measurement but also contributes to the design of culturally responsive, technology-enhanced interventions. Addressing these areas will help refine both the theoretical and practical dimensions of SEL in increasingly digital learning contexts.

## Data Availability

The datasets presented in this study can be found in online repositories. The names of the repository/repositories and accession number(s) can be found in the article/supplementary material.
