# Supplementary material for: Social–emotional architecture of adolescent success: a mixed-methods investigation of social–emotional learning pathways in Chinese secondary education
Source: Front Psychol. 2026 Jan 6;16:1739807. doi: 10.3389/fpsyg.2025.1739807 (PMC12816343; doi:10.3389/fpsyg.2025.1739807)
Supplement: Supplementary file 1 [file Table_1.DOCX]

**Appendix A**

**A questionnaire for qualitative research on the evaluation of adolescent social and emotional learning ability**

Dear students,

Hello! With the rapid development of society and the continuous innovation of education, the social and emotional learning ability of adolescents has received more and more attention. This questionnaire will focus on eight aspects to evaluate the social and emotional learning ability of adolescents. Your answers will not be ranked high or low, and all information will be strictly confidential and used only for scientific research. Thank you for your participation!

1. Your age:

A. 13 years old or younger B. 14 years old C. 15 years old D. 16 years old or older

2. Your gender:

A. Male B. Female

3. Your grade:

A. 7th grade (Junior 1) B. 8th grade (Junior 2) C. 9th grade (Junior 3)

D. 10th grade (Senior 1) E. 11th grade (Senior 2) F. 12th grade (Senior 3)

**Social Awareness and Interpersonal Relationships**

4. Please describe how you understand the emotions and needs of others.

5. Can you share how your social understanding abilities affect your learning style?

**Self-management and Personal Development**

6. How do you usually regulate your emotions and behaviors?

7. How would you evaluate your self-management abilities? How do these abilities help you in learning, interpersonal relationships, and avoiding negative behaviors (such as online addiction)?

**Interpersonal Pressure and Online Behavior**

8. Please describe the pressures you experience in interpersonal interactions.

9. Can you talk about your habits and patterns of internet use?

**Quality of Relationships and Stress Relief**

10. Please describe the characteristics of high-quality interpersonal relationships, in your opinion.

11. How do you handle stress in interpersonal relationships?

**Academic Factors and Decision-making Ability**

12. Please describe how you usually make decisions.

13. Can you share your study methods?

**Perception of Multilevel Relationships**

14. Please describe how your emotional state affects your academic performance.

15. How do you view the relationship between academic pressure and other aspects of life?

**Feedback on Intervention Strategies**

16. What type of support do you think is most helpful in reducing excessive internet use?

17. What kinds of resources would you like schools or institutions to provide to support student development?

**Verification of Integrated Perspectives**

18. Please describe the connections between different aspects of your life (social, emotional, academic, behavioral). 19. Can you share an example of how a change in one aspect has impacted other aspects?

**Appendix B**

**Adolescent Social-Emotional Learning Ability Assessment Questionnaire**

Dear students,

Hello! With the rapid development of society and the continuous innovation of education, the social and emotional learning ability of adolescents has received more and more attention. This questionnaire will focus on eight aspects to evaluate the social and emotional learning ability of adolescents. Your answers will not be ranked high or low, and all information will be strictly confidential and used only for scientific research. Thank you for your participation!

1. Your age:

A. 13 years old or younger B. 14 years old C. 15 years old D. 16 years old or older

2. Your gender:

A. Male B. Female

3. Your grade:

A. 7th grade (Junior 1) B. 8th grade (Junior 2) C. 9th grade (Junior 3)

D. 10th grade (Senior 1) E. 11th grade (Senior 2) F. 12th grade (Senior 3)

Each question has five options, ranging from "Strongly Disagree" to "Strongly Agree." Please choose the answer that best fits your situation.

4. When I decide upon a goal, I stick to it OR I can change a goal again at any time.

A. Totally Disagree B. Disagree C. Neither Agree nor Disagree D. Agree E. Totally Agree

5. When I do not succeed right away at what I want to do, I don’t try other possibilities for very long OR I keep trying as many different possibilities as are necessary to succeed at my goal.

A. Totally Disagree B. Disagree C. Neither Agree nor Disagree D. Agree E. Totally Agree

6. When I have started something that is important to me, but has little chance at success, I make a particular effort OR When I start something that is important to me but has little chance at success, I usually stop trying.

A. Totally Disagree B. Disagree C. Neither Agree nor Disagree D. Agree E. Totally Agree

7. Even if something is important to me, it can happen that I don’t invest the necessary time or effort OR For important things, I pay attention to whether I need to devote more time or effort.

A. Totally Disagree B. Disagree C. Neither Agree nor Disagree D. Agree E. Totally Agree

8. Sometimes I get mad at myself, but other times I am pretty pleased with myself.

A. Totally Disagree B. Disagree C. Neither Agree nor Disagree D. Agree E. Totally Agree

9. Sometimes I’m not very happy with the way I do things, but other times I think the way I do things is fine.

A. Totally Disagree B. Disagree C. Neither Agree nor Disagree D. Agree E. Totally Agree

10. Sometimes I don’t like the way I’m leading my life, but other times I do like the way I’m leading my life.

A. Totally Disagree B. Disagree C. Neither Agree nor Disagree D. Agree E. Totally Agree

11. Sometimes I get in trouble because of the things I do, but other times I don’t do things that get me in trouble.

A. Totally Disagree B. Disagree C. Neither Agree nor Disagree D. Agree E. Totally Agree

12. I care about how others feel and try to understand their thoughts.

A. Totally Disagree B. Disagree C. Neither Agree nor Disagree D. Agree E. Totally Agree

13. I don’t make up stories to avoid getting into trouble.

A. Totally Disagree B. Disagree C. Neither Agree nor Disagree D. Agree E. Totally Agree

14. I consider others’ feelings when interacting with them.

A. Totally Disagree B. Disagree C. Neither Agree nor Disagree D. Agree E. Totally Agree

15. I treat others with respect, regardless of their background or opinions.

A. Totally Disagree B. Disagree C. Neither Agree nor Disagree D. Agree E. Totally Agree

16. I am willing to assist classmates when they ask for support.

A. Totally Disagree B. Disagree C. Neither Agree nor Disagree D. Agree E. Totally Agree

17. Sometimes I find it hard to make friends, but other times I find it pretty easy.

A. Totally Disagree B. Disagree C. Neither Agree nor Disagree D. Agree E. Totally Agree

18. Sometimes I feel like I’m kind of hard to like, but other times I feel like I’m really easy to like.

A. Totally Disagree B. Disagree C. Neither Agree nor Disagree D. Agree E. Totally Agree

19. Sometimes I wish more people liked me, but other times I feel that most people do like me.

A. Totally Disagree B. Disagree C. Neither Agree nor Disagree D. Agree E. Totally Agree

20. I trust my friends.

A. Totally Disagree B. Disagree C. Neither Agree nor Disagree D. Agree E. Totally Agree

21. I feel my friends are good friends.

A. Totally Disagree B. Disagree C. Neither Agree nor Disagree D. Agree E. Totally Agree

22. My friends care about me.

A. Totally Disagree B. Disagree C. Neither Agree nor Disagree D. Agree E. Totally Agree

23. My friends are there when I need them.

A. Totally Disagree B. Disagree C. Neither Agree nor Disagree D. Agree E. Totally Agree

24. I want to help other people.

A. Totally Disagree B. Disagree C. Neither Agree nor Disagree D. Agree E. Totally Agree

25. I want to help make the world a better place to live in.

A. Totally Disagree B. Disagree C. Neither Agree nor Disagree D. Agree E. Totally Agree

26. I am willing to give time and money to make life better for other people.

A. Totally Disagree B. Disagree C. Neither Agree nor Disagree D. Agree E. Totally Agree

27. I want to help reduce hunger and poverty in the world.

A. Totally Disagree B. Disagree C. Neither Agree nor Disagree D. Agree E. Totally Agree

28. I want to help make sure all people are treated fairly.

A. Totally Disagree B. Disagree C. Neither Agree nor Disagree D. Agree E. Totally Agree

29. I am willing to speak up for equality (everyone should have the same rights and opportunities).

A. Totally Disagree B. Disagree C. Neither Agree nor Disagree D. Agree E. Totally Agree

30. I feel that the daily pressure of school causes me to have difficulty balancing my studies with other activities (e.g., sports, recreation).

A. Totally Disagree B. Disagree C. Neither Agree nor Disagree D. Agree E. Totally Agree

31. I find the amount of homework too much to cope with.

A. Totally Disagree B. Disagree C. Neither Agree nor Disagree D. Agree E. Totally Agree

32. The frequency of tests stresses me out because there is a test or quiz almost every week.

A. Totally Disagree B. Disagree C. Neither Agree nor Disagree D. Agree E. Totally Agree

33. I would feel a lot of stress from worrying about my grades.

A. Totally Disagree B. Disagree C. Neither Agree nor Disagree D. Agree E. Totally Agree

34. I often Feel low in energy or slowed down

A. Totally Disagree B. Disagree C. Neither Agree nor Disagree D. Agree E. Totally Agree

35. I cry easily.

A. Totally Disagree B. Disagree C. Neither Agree nor Disagree D. Agree E. Totally Agree

36. I feel blue.

A. Totally Disagree B. Disagree C. Neither Agree nor Disagree D. Agree E. Totally Agree

37. I feel easily annoyed or irritated.

A. Totally Disagree B. Disagree C. Neither Agree nor Disagree D. Agree E. Totally Agree

38. Temper outbursts that you could not control.

A. Totally Disagree B. Disagree C. Neither Agree nor Disagree D. Agree E. Totally Agree

39. I have urges to beat, injure, or harm someone.

A. Totally Disagree B. Disagree C. Neither Agree nor Disagree D. Agree E. Totally Agree

40. I often lose my appetite.

A. Totally Disagree B. Disagree C. Neither Agree nor Disagree D. Agree E. Totally Agree

41. I often have trouble sleeping.

A. Totally Disagree B. Disagree C. Neither Agree nor Disagree D. Agree E. Totally Agree

42. I have thoughts of death or dying.

A. Totally Disagree B. Disagree C. Neither Agree nor Disagree D. Agree E. Totally Agree

43. I binge eat a lot.

A. Totally Disagree B. Disagree C. Neither Agree nor Disagree D. Agree E. Totally Agree

44. Sometimes other children are unkind to me.

A. Totally Disagree B. Disagree C. Neither Agree nor Disagree D. Agree E. Totally Agree

45. I’m easily hurt by what others say about me.

A. Totally Disagree B. Disagree C. Neither Agree nor Disagree D. Agree E. Totally Agree

46. I find it difficult to make new friends.

A. Totally Disagree B. Disagree C. Neither Agree nor Disagree D. Agree E. Totally Agree

47. My parents has argued with me.

A. Totally Disagree B. Disagree C. Neither Agree nor Disagree D. Agree E. Totally Agree

48. My parents has punished me too hard.

A. Totally Disagree B. Disagree C. Neither Agree nor Disagree D. Agree E. Totally Agree

49. I have been of no importance to my parents.

A. Totally Disagree B. Disagree C. Neither Agree nor Disagree D. Agree E. Totally Agree

50. My parents has been vary anxious about me.

A. Totally Disagree B. Disagree C. Neither Agree nor Disagree D. Agree E. Totally Agree

51. When reading for this class, I try to relate the material to what I already know.

A. Totally Disagree B. Disagree C. Neither Agree nor Disagree D. Agree E. Totally Agree

52. When I study for this course, I go over my class notes and make an outline of important concepts.

A. Totally Disagree B. Disagree C. Neither Agree nor Disagree D. Agree E. Totally Agree

53. I make sure that I keep up with the weekly readings and assignments for this course.

A. Totally Disagree B. Disagree C. Neither Agree nor Disagree D. Agree E. Totally Agree

54. Even when course materials are dull and uninteresting, I manage to keep working until I finish.

A. Totally Disagree B. Disagree C. Neither Agree nor Disagree D. Agree E. Totally Agree

55. When I can’t understand the material in this course, I ask another student in this class for help.

A. Totally Disagree B. Disagree C. Neither Agree nor Disagree D. Agree E. Totally Agree

56. Do you find that you stay on-line longer than you intended?

A. Totally Disagree B. Disagree C. Neither Agree nor Disagree D. Agree E. Totally Agree

57. Do others in your life complain to you about the amount of time you spend on-line?

A. Totally Disagree B. Disagree C. Neither Agree nor Disagree D. Agree E. Totally Agree

58. Does your work suffer (e.g. postponing things, not meeting deadlines, etc.) because of the amount of time you spend on-line?

A. Totally Disagree B. Disagree C. Neither Agree nor Disagree D. Agree E. Totally Agree

59. Do you snap, yell, or act annoyed if someone bothers you while you are on-line?

A. Totally Disagree B. Disagree C. Neither Agree nor Disagree D. Agree E. Totally Agree

60. Do you lose sleep due to late night logins?

A. Totally Disagree B. Disagree C. Neither Agree nor Disagree D. Agree E. Totally Agree

61. People can count on me to keep on schedule.

A. Totally Disagree B. Disagree C. Neither Agree nor Disagree D. Agree E. Totally Agree

62. I am always on time.

A. Totally Disagree B. Disagree C. Neither Agree nor Disagree D. Agree E. Totally Agree

63. I am reliable.

A. Totally Disagree B. Disagree C. Neither Agree nor Disagree D. Agree E. Totally Agree
